# Remote sensing appraisal of Lake Chad shrinkage connotes severe impacts on green economics and socio-economics of the catchment area

**DOI:** 10.1098/rsos.171120

**Published:** 2017-11-08

**Authors:** Olapeju Y. Onamuti, Emmanuel C. Okogbue, Israel R. Orimoloye

**Affiliations:** 1 Department of Meteorology and Climate Science, School of Earth and Mineral Sciences, Federal University of Technology Akure, PMB 704, Akure, Nigeria; 2 Department of Geography and Environmental Science, School of Agriculture and Science, University of Fort Hare, Alice 5700, South Africa

**Keywords:** remote sensing, shrinkage, land use, ecological sustainability, socio-economics, climate change

## Abstract

Lake Chad commonly serves as a major hub of fertile economic activities for the border communities and contributes immensely to the national growth of all the countries that form its boundaries. However, incessant and multi-decadal drying via climate change pose greater threats to this transnational water resource, and adverse effects on ecological sustainability and socio-economic status of the catchment area. Therefore, this study assessed the extent of shrinkage of Lake Chad using remote sensing. Landsat imageries of the lake and its surroundings between 1987 and 2005 were retrieved from Global Land Cover Facility website and analysed using Integrated Land and Water Information System version 3.3 (ILWIS 3.3). Supervised classification of area around the lake was performed into various land use/land cover classes, and the shrunk part of its environs was assessed based on the land cover changes. The shrinkage trend within the study period was also analysed. The lake water size reduced from 1339.018 to 130.686 km^2^ (4.08–3.39%) in 1987–2005. The supervised classification of the Landsat imageries revealed an increase in portion of the lake covered by bare ground and sandy soil within the reference years (13 490.8–17 503.10 km^2^) with 4.98% total range of increase. The lake portion intersected with vegetated ground and soil also reduced within the period (11 046.44–10 078.82 km^2^) with 5.40% (967.62 km^2^) total decrease. The shrunk part of the lake covered singly with vegetation increased by 2.74% from 1987 to 2005. The shrunk part of the lake reduced to sand and turbid water showed 5.62% total decrease from 1987 to 2005 and a total decrease of 1805.942 km^2^ in area. The study disclosed an appalling rate of shrinkage and damaging influences on the hydrologic potential, eco-sustainability and socio-economics of the drainage area as revealed using ILWIS 3.3.

## 1. Introduction

In recent years, there are alarming reports of phenomenal contraction and total disappearance of many freshwaters and wetlands in the world. Retractions and extinctions of waterbodies are primarily connected to climate change, hydro-climatological pressures and anthropological workouts [1–6]. Of concern are lakes and their catchment ecosystems, due to the various importance they serve. Shrinkage of lakes is not principally of local concern, but also a matter of regional and global attentions [7]. The rapid damaging impacts on sustainability of environmental green economics and deterioration of socio-economic status in the retracting lake basins call for priority conservational efforts. For instance, diminution of many African lakes [8,9] and accompanying paralysed livelihoods, food shortage, disease outbreaks, high penury rates, violence outbreaks, conflicts, terror attacks and social insecurity etc. in lakeshores/catchments have been reported [10–15]. Hence, various research enterprises have been channelled at monitoring and assessing the extent of changes in lakes and their eco-environmental status to alert policymakers towards precautionary activities and plans for future water resources. In addition, informational needs for future land use, wetland managements, political decisions and activities, and water management are essentials for which many investigations were conducted [5,15,16].

Lake Chad is one of the most important transnational and transboundary freshwater resources in the world. It occupies 8% of African continental land area. The lake expanse extends to eight African nations involving Cameroon, Algeria, Chad, Central African Republic, Niger, Libya, Sudan and Nigeria [17]. The lake is geographically located within the Sudano-Sahelian region, at the coordinates 12°20 N–14°20 N and 3°00 E–15°20 E [18]. The historic sixth largest lake of the world has been documented as continuously experiencing desiccation and loss of areal coverage in past decades [19,20]. Goni *et al*. [21] recently presented a review of historical climate viability and areal retreat of Lake Chad basin. Various shrinkages in Lake Chad reported by earlier investigators include shift from 13 000 to 26 000 km^2^ [22], approximately 88% areal decline [23], approximately 95% extent loss in 1963–2000 [24]. Adewuyi [23] also estimated 56% increase in the lake extent between 1997 and 2001. More so, Ebenki [24] found that the lake fluctuated in size in 1975–1990 by 15% increase and in 2000–2007 with 11% reduction. Noteworthy, Ebenki [24] recorded 15% increase in the lake area between 1975 and 1990, 9% decline between 1990 and 2000 and approximately 11% areal decline in 2000–2007. Areal drifts or shift in Lake Chad extent in the 1960s–1970s and in the 1980s–2000s were presented as 25 000 km^2^ and 3000–7000 km^2^, respectively [17,21].

Previous studies on Lake Chad and vicinity employed instrumentation of direct site survey including interviews, questionnaires and photographs [15]. Besides that these methods are backbreaking, time depleting and cost-ineffective, they involve low coverage because information about inaccessible and remote areas of the lake is usually excluded. Investigations using remote sensed dataset or satellite imageries have also been reported on the lake [24–26]. Remote sensing provides an excellent environment for cost-effective mapping, cheap imageries, geographical information system, that allow efficient monitoring, areal change detection and modelling of inaccessible natural resources, environmental variables and phenomena [26–28]. Remote sensed imageries and approaches not only serve as powerful tools for information collection, processing and management in inaccessible and hostile environment, hydro-meteorological events such as hurricanes, floods, land cover/land use and environmental scars, but also they are effective communication tools [26,29,30].

Nevertheless, the effective, efficient and accurate application of remote sensing in assessment of areal extent of lakes and other freshwater resources is largely not independent of the dataset, data source and instrumentation. For illustration, Adewuyi [23] employed Modis, Landsat and Argon imageries between 1963 and 2001 (dataset for 1963, 1973, 1987, 1997 and 2001); and GIS tools including Arc view 3.1 and Arc map 8.1 in his investigation of Lake Chad. Similarly, Alfa *et al*. [25] used satellite imageries for 1963, 1972, 1987 and 2000, and ERDAS Imagine environment for the lake. Landsat MSS (1975), Landsat TM (1990), Landsat ETM+ (2000), ASTER (2007), satellite imagery from the Terralook database and GIS techniques were adopted by Ebenki [24]. NigeriaSat-1 and Landsat dataset have also been applied in classification of the land use/land cover of the area [26]. However, these various studies output dissimilar results. Further remote sensing procedures and instrumentation need to be assessed in order to compare suitability of the different approaches in assessment of Lake Chad extent contraction. Therefore, the present study appraised Lake Chad shrinkage using remote sensing and instrumentation of Integrated Land and Water Information System v. 3.3 (ILWIS 3.3). This was the first investigation that analysed remote sensed dataset (Landsat imageries) of the lake and its surroundings using ILWIS 3.3. The study monitored shrinkage and reduction in Lake Chad using satellite images. Firstly, supervised classification of the satellite images of the Lake Chad basin was done into various land use/land cover classes for the reference years (1987–2005), secondly land cover extent of the various land cover classes was determined, and finally the shrinkage pattern within the study period was computed.

## 2. Methodology

### 2.1. The study area

**Figure 1.**
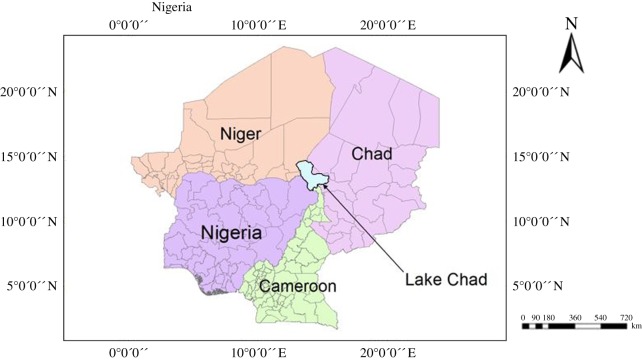
Location of the study area (Lake Chad) on Nigeria map.

The ‘standard’ Lake Chad is situated between latitudes 12°10′ N and 14°30′ N, and longitudes 13° E and 15°30′ E in the hot semi-arid region. The lake is located along the international boundary of four countries which are Nigeria, Niger, Chad and Cameroon, and it is the fourth largest lake in Africa. Its hydrological basin (2.4 million km^2^ extent) constitutes freshwater source shared by eight African countries which are Niger, Cameroon, Nigeria, Chad Republic, Central African Republic, Sudan, Libya and Algeria. It is fed by Chari-Logone river systems from the south and Komadugu/Yobe-Ngadda river systems from the western part of the lake. The catchment area occupies approximately 2 434 000 km^2^ (approx. 8%) of African total continental land surface [17]. It is situated on an altitudal plateau of estimated 283 m above average sea level [31] (figure 1).

### 2.2. Data and software

Landsat imageries and ILWIS 3.3 of the lake and its environs for years 1987–2005 were acquired from Global Land Cover Facility website (http://glcfapp.glcf.umd.edu/) and the National Space Research and Development Agency, Nigeria, respectively. The data attributes are presented in table 1. ILWIS software (ILWIS 3.3) was acquired from Strategic Space Department of National Space Research and Development Agency (NASRDA), Nigeria.

**Table 1.** Specification of data used.

data	year	bands
Landsat thematic mapper (Landsat5 TM)	1987	2, 3, 4
Landsat enhanced thematic mapper plus (Landsat7 ETM+)	1999	2, 3, 4
Landsat enhanced thematic mapper plus (Landsat7 ETM+)	2005	2, 3, 4

### 2.3. ILWIS version 3.3 data analysis

#### 2.3.1. Band combination

The Landsat data in raster formats or TIFF formats were subjected to ILWIS 3.3 analysis for band combination to obtain a pictorial image of the study area. Band combination was performed on the basis that each specific wavelength range was stored as a separate image (band). The band combination of satellite images was carried out in order of red, green and blue.

#### 2.3.2. Image classification, interpretation and embellishment

The pictorial image or general overview (features) of the location obtained from band combination was further subjected to supervised classification using ILWIS 3.3. This was done to categorize physical (Earth) characteristics feature of the study location. Unlike unsupervised classification whereby the software picks the characteristics features (points) of the images and places them in categories without specific name, the supervised classification involves a human operator deciding the points or area of the combined image to be categorized with specific name tag. The land cover classes considered in the supervised classification include (i) current water in the lake, (ii) sandy soil and bare ground, (iii) the shrunk part of the lake covered with vegetation, (iv) the shrunk part of the lake covered with soil and vegetation, and (v) the shrunk part of the lake reduced to sand and turbid water. The knowledge of correct band combination helps in the interpretation of the already classified image. The classified image was then embellished by adding appropriate layout (north arrow, title bar, legend, scale, text and other features). Also, comparison plots of the years were performed.

In all cases, the number of pixels corresponding to a specific land cover class was determined with raster calculator. Then, corresponding coverage area was estimated through cross-multiplication of its number of pixels in the attribute table and the image resolution.

## 3. Results and analysis

### 3.1. Land use features of Lake Chad from 1987 supervised classified image

**Figure 2.**
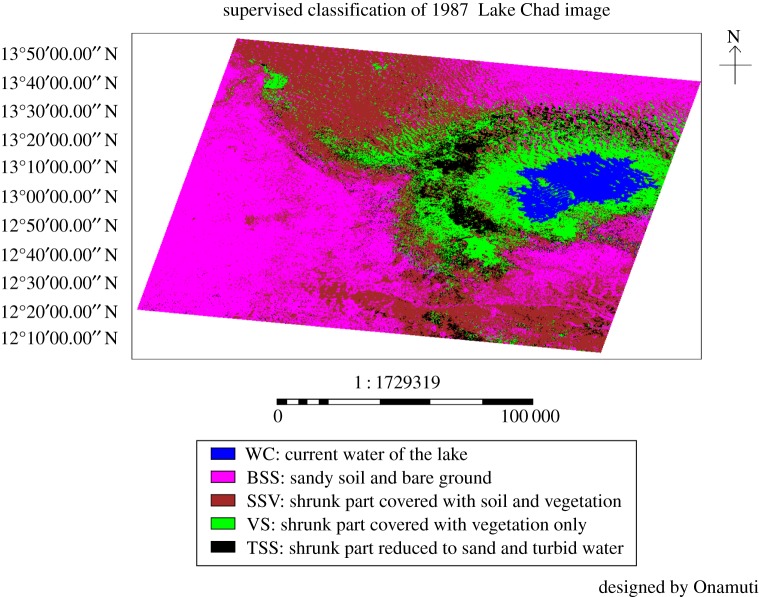
The supervised classification of Lake Chad and its environs for the year 1987.

The supervised classification of Lake Chad and its environs for year 1987 is presented in figure 2, while the corresponding area coverage for the various land use/land cover identified is presented in table 2. The area coverage of the current water in the lake as at 1987 was 1339.018 km^2^. The portion covered by sandy soil and bare ground was 13 490.8 km^2^. While the shrunk part covered with soil and vegetation was 11 046.44 km^2^, the shrunk area vegetated only was 4610.831 km^2^. A 2306.929 km^2^ area of the shrunk part was reduced to sand and turbid water at 1987.

**Table 2.** The area coverage of the 1987 supervised classification image.

various land use/land cover identified	no. pixels	area coverage (km^2^)	percentage coverage (%)
current water of the lake	1 648 530	1339.018	4.08
sandy soil and bare ground	16 609 170	13 490.8	41.14
shrunk part covered with soil and vegetation	13 599 807	11 046.44	33.68
shrunk part covered with vegetation only	5 676 616	4610.831	14.06
shrunk part reduced to sand and turbid water	2 840 171	2306.929	7.03

### 3.2. Land use features of Lake Chad from 1999 supervised classified image

**Figure 3.**
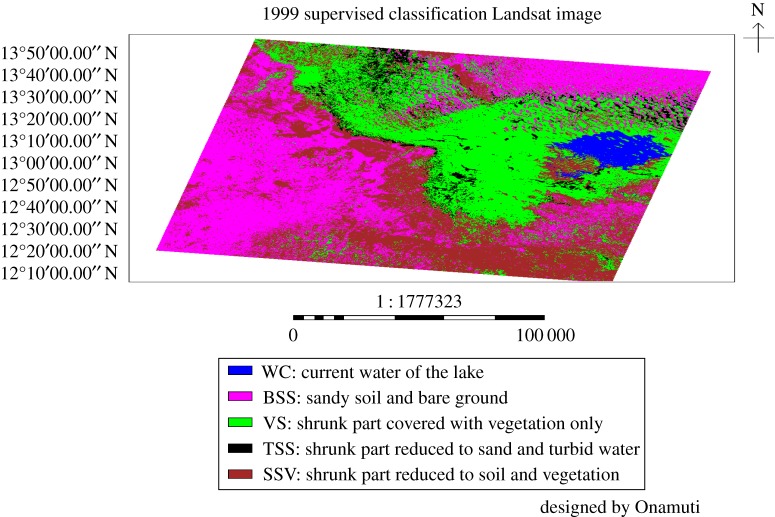
The supervised classification of Lake Chad and its environs for the year 1999.

Figure 3 depicts the physical attributes of Lake Chad from the 1999 supervised classification image. The corresponding area coverage for various land use of the lake and environs for the year is also presented in table 3. At the year 1999, Lake Chad had an area coverage of 876.2975 km^2^ (2.6%) for current water. Sandy soil and bare ground covered 11 486.33 km^2^ (34.14%). The shrunk part of the lake reduced to vegetation only covered 9362.569 km^2^ (27.83%). Approximately, 4.17% (1402.323 km^2^) of the shrunk portion of the lake was reduced to sand and turbid water at year 1999.

**Table 3.** The area coverage for different land cover of the 1999 supervised classification image.

various land use/land cover identified	no. pixels	area coverage (km^2^)	percentage land cover (%)
current water of the lake	1 078 852	876.2975	2.6
sandy soil and bare ground	14 141 374	11 486.33	34.14
shrunk part covered with vegetation only	11 526 709	9362.569	27.83
shrunk part reduced to sand and turbid water	1 726 467	1402.323	4.17

### 3.3. Land use features of Lake Chad from 2001 supervised classified image

**Figure 4.**
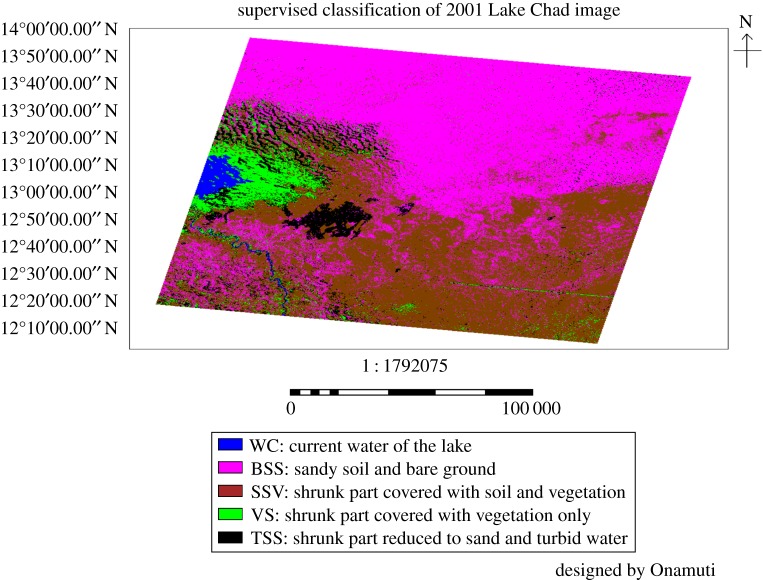
The supervised classification of Lake Chad and its environs for the year 2001.

The 2001 supervised classification image of Lake Chad basin is presented in figure 4 and the associated coverage area in table 4. The classification image showed only a part of the lake and not the entire catchment area due to unavailability of the complete imagery and high percentage of cloud cover. The 2001 supervised classified image of the lake was excluded from further analysis in this study.

**Table 4.** The area coverage for different land cover of the 2001 supervised classification image.

various land use/land cover identified	no. pixels	area coverage (km^2^)
current water of the lake	452 458	367.509
sandy soil and bare ground	20 116 231	16 339.41
shrunk part covered with soil and vegetation	16 302 475	13 241.69
shrunk part covered with vegetation only	2 011 726	1634.024
shrunk part reduced to sand and turbid water	2 676 654	2174.112

### 3.4. Land use features of Lake Chad from 2005 supervised classified image

**Figure 5.**
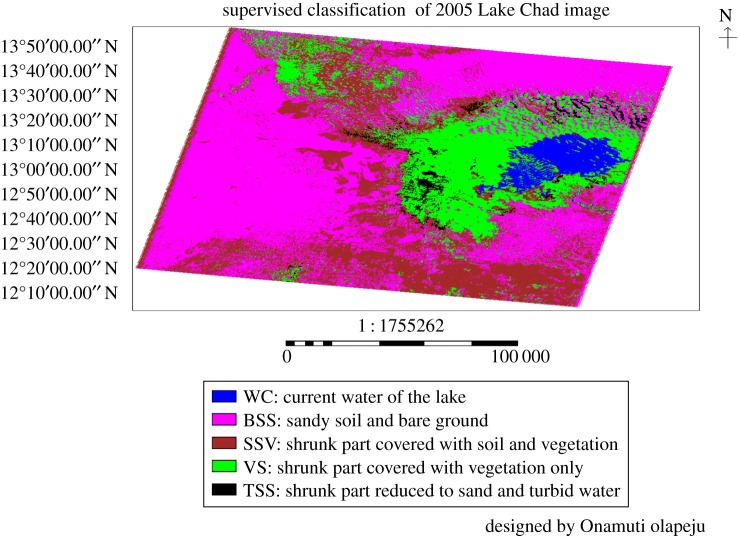
The supervised classification of Lake Chad and its environs for the year 2005.

The physical characteristics of Lake Chad and its environs in the year 2005 are shown in the supervised classification in figure 5. Land use coverage area of certain identified classes at the year is presented in table 5. The year 2005 presented 1208.332 km^2^ (3.39%) area coverage attributed to current water in the lake. While sandy soil and bare ground occupied 17 503.1 km^2^ (49.12%) land coverage area. The shrunk part of the lake covered with vegetated soil was 10 078.83 km^2^ (28.28%). The shrunk area of the lake covered solely with vegetation was 6342.145 km^2^ (17.8%). A 500.988 km^2^ (1.41%) area of the shrunk part of the lake was reduced to sand and turbid water at 2005.

**Table 5.** The area coverage for different land cover of the 2005 supervised classification image.

various land use/land cover identified	no. pixels	area coverage (km^2^)	percentage land cover (%)
current water of the lake	1 342 591	1208.332	3.39
sandy soil and bare ground	19 447 893	17 503.1	49.12
shrunk part covered with soil and vegetation	11 198 697	10 078.83	28.28
shrunk part covered with vegetation only	7 046 828	6342.145	17.8
shrunk part reduced to sand and turbid water	556 653	500.988	1.41

### 3.5. The compared area coverage of different land cover classes for the study period (1987, 1999, 2005)

The compared land cover classes for the reference years 1987, 1999 and 2005 are shown in table 6. The current water in the lake at the years 1987, 1999 and 2005 has a coverage area of 1339.018 km^2^ (4.08%), 876.2975 km^2^ (2.6%) and 1208.332 km^2^ (3.39%), respectively. Similarly, the respective coverage area of sandy soil and bare ground was 13 490.8 km^2^ (41.14%), 11 486.33 km^2^ (34.14%) and 17 503.104 km^2^ (49.12%) for the years 1987, 1999 and 2005. The shrunk portion of the lake associated with soil and vegetation has area coverage of 11 046.44 km^2^ (33.68%), 10 519.73 km^2^ (31.26%) and 10 078.827 km^2^ (28.28%) in the years 1987, 1999 and 2005, respectively. Area coverage of 4610.831 km^2^ (14.06%), 9362.569 km^2^ (27.83%) and 6342.145 km^2^ (17.8%) accounted for shrunk part of the lake covered with vegetation only in the years 1987, 1999 and 2005, respectively. The shrunk area of the lake reduced to sand and turbid water was 2306.929 km^2^ (7.03%) in 1987, 1402.323 km^2^ (4.17%) in 1999 and 500.988 km^2^ (1.41%) in 2005.

**Table 6.** The compared area coverage of various land cover classes for a 3 year time-series (figures 2, 3 and 5). The percentage average area coverage of the different land cover classes is presented in parentheses against each class.

	area covered by the shrinking lake and its surroundings at different years (km^2^)		
various land use/land cover classes	1987 (%)	1999 (%)	2005 (%)
current water in the lake	1339.018 (4.08)	876.2975 (2.6)	1208.332 (3.39)
sandy soil and bare ground	13 490.8 (41.14)	11 486.33 (34.14)	17 503.104 (49.12)
shrunk part covered with soil and vegetation	11 046.44 (33.68)	10 519.73 (31.26)	10 078.827 (28.28)
shrunk part covered with vegetation only	4610.831 (14.06)	9362.569 (27.83)	6342.145 (17.8)
shrunk part reduced to sand and turbid water	2306.929 (7.03)	1402.323 (4.17)	500.988 (1.41)

## 4. Discussion

The application of remote sensing via ILWIS 3.3 instrumentation to hydro-climatic phenomena associated with Lake Chad catchment area was carried out in this study. Owing to the merits that remote sensing allows vivid assessment of the extent of changes and estimate of accompanying earth physical characteristics, hydro-ecologic to socio-economic manifestations, the various changes in the lake extent and land cover classes of its environs were assessed. Remote sensing provides synoptically repeated observations, frequent wetland maps, surrounding land uses attributes and time-serial changes, which overcome limitations posed by spatial and temporal coverage in conventional measurements (field surveys/gauge stations) in wetland monitoring [32–34].

### 4.1. Current water in the lake

This is the portion of the supervised classification of the Landsat imageries of Lake Chad for the individual year that depicts the real extent of water present in the lake (i.e. 1987, 1999 and 2005). In other words, it is the Earth surface that the lake presently covers due to its shrinkage in each reference year. The reduction in the lake water coverage area and extent from 1339.018 km^2^ in 1987 to 876.297 km^2^ in 1999, and a total size decrease of 130.686 km^2^ from 1987 to 2005 (table 6), could be attributed to both climatic and anthropogenic influences. Anthropogenic demands and climatic variability have been associated with the induced and accelerated eco-environmental changes in the lake catchment area [21]. Climatic phenomena such as high evaporation and low precipitation are reported contributors to severe lake shrinkage [35–37]. The mean annual evaporation rate (1600 mm) of the lake basin previously reported was twofold higher in magnitude compared to its average annual rainfall rate (approx. 625 mm) [38]. Furthermore, annual maximum temperatures of the lake ranged between 35 and 40°C, especially in the northern catchment area [39] with annual average temperature of 21.4°C [8].

The lake extent or current water size obtained in this study showed discordant values to estimates in other studies [22,23,25]. However, the results fell within the range and followed similar patterns to those reported. This could probably be due to differences in dataset used, study periods and instrumentation. For instance, Adewuyi [23] employed Modis, Landsat and Argon imageries between 1963 and 2001 (dataset for 1963, 1973, 1987, 1997 and 2001), and GIS tools including Arc view 3.1 and Arc map 8.1 in his investigation. Similarly, Alfa *et al*. [25] used satellite imageries for 1963, 1972, 1987 and 2000, and ERDAS Imagine environment. Also, Landsat MSS (1975), Landsat TM (1990), Landsat ETM+ (2000), ASTER (2007), satellite imagery from the Terralook database [40] and GIS techniques were adopted by Ebenki [24]. Ebenki [24] in addition used the *K*-means unsupervised classification in contrast with the supervised classification employed in this study. Thus, the supervised classification used in this study overcame possible classification of homogeneous spectra classes within the dataset which necessarily do not equal the same information family, unlike misclassification that commonly accompanies unsupervised classification [24,41].

Certain estimated shrinkage in Lake Chad reported in the literature includes 13 000–26 000 km^2^ [22], 40 000–4837 km^2^ from 1963 to 1997, an approximately 88% areal size [23] and 20 900–304 km^2^, an approximately 95% extent loss in 1963–2000 [24]. This present study observed fluctuation in the area extent of the lake, depicted as 4.08% decrease in 1987 to 2.6% in 1999 and then to 3.39% in 2005. This agrees with Adewuyi [23], who estimated 56% increase in the lake extent between 1997 and 2001. Also, Ebenki [24] estimated that the lake fluctuated in size in 1975–1990 between 8065 and approximately 12 813 km^2^ (a 15% extent increase) and a decline in 2000–2007 from 10 011 to approximately 8251 km^2^ (11% extent reduction). Generally, Ebenki [24] noted 15% increase in the lake area extent between 1975 and 1990, 9% extent decline between 1990 and 2000 and approximately 11% area decline in 2000–2007.

### 4.2. Bare ground and sandy soil

This is the portion of the supervised classified Landsat images of Lake Chad for individual year (1987, 1999 and 2005) that depicts its surrounding covered by sandy soil and the ground surface only (table 6). The increase in this land cover class from 13 490.8 km^2^ in 1987 to 17 503.10 km^2^ in 2005, and with a range of 6016.774 km^2^, could be due to incessant abstraction of the lake for various purposes, desertification and frequent drought experienced in the basin. Drought conditions have been linked to increase in bare ground around lakes and diminished surface extent [21,26,42]. Conventionally, prolonged droughts are frequently connected with dune or bare ground formation in lake and other wetland areas [21]. Oftentimes, there is a general inclination towards dune establishment and major changes in lake hydrology and vegetation, provided there is sufficient wind energy [21]. Fluctuations in the total area coverage by bare ground and sandy soil from 41.14% in 1987 to 34.14% in 1999 (decline trend), and from 34.14 to 49.12% in 1999 and 2005 (increase trend), could be connected to alternating encroachment, afforestation/agricultural activities and deforestation of the lake environment. Increasing farmland and livestock density, and overgrazing beyond the carrying capacity of the lake's grassland could additionally contribute to bare land formation [43]. A total range of 4.98% increase in the lake extent covered by bare ground and sandy soil obtained in this study is not without its attending negative consequences on the lake ecology and socio-economics of the drainage area. This could be manifested as reduction in farmable land area, dwindling food and animal feed resources, loss of fishing ground, water inaccessibility, forced migrations and resettlement, competition, conflict among settlers, recession farming, transboundary activities and national tension [21,43].

### 4.3. Shrunk part of the lake covered with soil and vegetation

This is the portion of the supervised classification of the Landsat images of Lake Chad intersected with a combination of vegetated ground and soil for individual year (1987, 1999 and 2005) (table 6). This land cover is primarily dominated by shrubs and fuel. A reduction in this areal extent from 11 046.44 km^2^ (33.68%) in 1987 to 10 078.82 km^2^ (28.28%) in 2005, totalling a 967.62 km^2^ (5.40%) decrease in the area, could generally be adduced to over-exploitation of wood and hydro-climatic forces. Eco-pastoral behaviours and grazing activities are potential linkages connected to the areal extent reduction in vegetated soil and ground coverage around the lake. Extensive bush burning, and deforestation, are other contributors to reduction in vegetated soil around the lake basin [44]. The disappearance of vegetated ground has ecologic effects on animal habitants. Game usually show migratory tendencies towards dense vegetated areas of the lake to seek shelter, and thus induce ecological imbalances in the lake ecosystem. Aggressive reactions and violence against intruders in game population could increase in species defence of their territories. Also, there is accompanying loss of both animal and plant biodiversity due to decrease in the lake vegetated soil and ground areal extent. Population shifts among human settlers towards farmable land area of the lake due to loss of fertile vegetated ground could be noticed. Southward migration among human settlers in search of grazing land, fertile farmland and irrigatable land has been documented along Lake Chad catchment for several years [21]. This has also resulted in degradation of the southern drainage area due to increased pressure on the available water resources, tree felling and livestock treading down of plants. Regional conflict and tension are other socio-economic factors.

### 4.4. Shrunk part of the lake covered with vegetation only

This is the portion of the supervised classification of the Lake vicinity covered by vegetation in the years 1987, 1999 and 2005 (table 6). This area is not intersected by sandy soil, but immediately next to the current water in the lake. The increase in the areal extent cover solely with vegetation from 4610.83 km^2^ (14.06%) in 1987 to 9362.57 km^2^ (27.83%) in 1999, and then a decline to 6342.15 km^2^ (17.8%), could depict possible precipitation pattern and interaction of various influences. Generally, a total of 2.74% areal extent increase in the shrunk part of the lake covered by vegetation from 1987 to 2005 could be attributed to hydro-climatological variables including water level and precipitation that might modulate the lake biomass and areal vegetation. Ecological succession, natural disturbances, impoundment and damming of inflow tributaries are other factors that could contribute to areal variability of lake vegetation [34].

### 4.5. Shrunk part of the lake reduced to sand and turbid water

This is area extent of the supervised classification of Lake Chad neighbourhood in 1987, 1999 and 2005 reduced to combination of eroded sand dunes, unclean or unclear pools of water. This part is in no way regarded as part of the present water in Lake Chad (table 6). The 5.62% (1805.942 km^2^) total decrease in the areal extent of the shrunk lake reduced to sand dune and turbid water from 1987 to 2005 could be ascribed to increasing successional colonization of the area by wetland plant species. Exposure of the zone due to contraction in lake water could lead to active germination of dormant lakeshore plant seeds and consequent regeneration of the region [45]. Increased lake water turbidity has been linked to direct modulation of submersed light, suppression of subaqueous vegetation and productivity, zonal stock reduction and senescence of fish/shrimp and threats to migratory bird habitats [45–48].

Other contributors to Lake Chad shrinkage include lake water discharge to groundwater, and dam construction on the tributaries. For instance, 45% water inflow reduction to the lake has been attributed to Tiga and Challawa dams constructed on Komadugu Yobe river in 1974 and 1992, respectively [21]. Also, 42%, approximately 60% and approximately 83% inflow reduction into the lake have been linked to Chari River, Kumadugu Yobe and El Beid, respectively, over the last 50 years [21]. Socio-economic outcome of the lake contraction could be evidenced as reduced crop yield and production, food and feed insecurity, biodiversity decline and loss, loss of fishing ground, increasing unemployment, increasing relocation and migration, high crime rate, and hunger and diseases [15].

## 5. Conclusion

The study revealed an alarming rate of Lake Chad contraction and damaging effects on the hydrologic potential, eco-sustainability and socio-economics of the drainage area. Also, ILWIS 3.3 showed a comparable utility in assessing hydro-meteorological events associated with freshwaters and wetlands. Its outputs concordantly fall within the same range and display similar patterns to other remote sensing instrumentations.

Therefore, the present study appraised Lake Chad shrinkage using remote sensing and instrumentation of ILWIS 3.3. This was the first investigation that analysed remote sensed dataset (Landsat imageries) of the lake and its surroundings using ILWIS 3.3.

## Data accessibility

Landsat imageries and ILWIS 3.3 of the lake and its environs for this study were acquired from Global Land Cover Facility website (http://glcfapp.glcf.umd.edu/) and the National Space Research and Development Agency, Nigeria, respectively. The data attributes are presented in table 1. ILWIS 3.3 was acquired from NASRDA, Nigeria.
